# Sewage of Baltimore, and the National Board of Health

**Published:** 1880-12

**Authors:** 


SEWAGE OF BALTIMORE, AND THE NATIONAL BOARD OF HEALTH.—We learn from the secular press that the National Board of Health, located in the city of Washington, has assumed to interfere with the hygenic management of our municipality by making certain reports and volunteering advice in regard to an extended and expensive system of sewers for Baltimore: and by publishing the same to to the world has produced an impression in regard to the sanitary condition of the city at variance with the facts as they are known to exist. We were originally quite favorably impressed with the creation by Congress of a Board of Health, that should have the officers and the means at its command to investigate the origin of yellow fever, and learn all that can be known of the laws governing its epidemical visitations to our cities, and thereby aid the municipal and state authorities in such prophylactic measures as might be necessary against the introduction of that disease. Beyond this, its powers should not be permitted to extend, save probably, to render such aid in supplying acclimated physicians and nurses, and affording such sanitary assistance as its resources might enable it to supply, during the actual existence of an epidemic; unless to perform the duties already adverted to in case of an expected visitation of cholera, or other *contagious* or infectious disease! Baltimore is unquestionably *one of the healthiest cities* in the United States, as statistics will clearly show, and therefore the report imputed to the National Board of Health, by newspapers, is calculated to produce a false impression in regard to its sanitary condition, and consequently to work serious injury to its prosperity and commerce. We are fortunately not yet an Empire and consequently, an obliteration of state lines, and the absorption of state and municipal authority by *any* body, has not yet been our lot. Such being the case, Baltimore ought to be able to take care of the health of her people, and will doubtless adopt such systems of sewage and *other* hygienic measures, as her municipal authorities may see fit to inaugurate under the direction of her own efficient health department.

